# Patient-Centered mHealth Living Donor Transplant Education Program for African Americans: Development and Analysis

**DOI:** 10.2196/resprot.3715

**Published:** 2015-08-10

**Authors:** John Christopher Sieverdes, Lynne S Nemeth, Gayenell S Magwood, Prabhakar K Baliga, Kenneth D Chavin, Brenda Brunner-Jackson, Sachin K Patel, Kenneth J Ruggiero, Frank A Treiber

**Affiliations:** ^1^ Technology Applications Center for Healthful Lifestyles College of Nursing Medical University of South Carolina Charleston, SC United States; ^2^ College of Nursing Medical University of South Carolina Charleston, SC United States; ^3^ Division of Transplant Surgery Department of Surgery, College of Medicine Medical University of South Carolina Charleston, SC United States; ^4^ Psychiatry Institute Ralph H. Johnson VA Medical Center Charleston, SC United States; ^5^ College of Medicine Medical University of South Carolina Charleston, SC United States

**Keywords:** kidney transplantation, living donors, mobile apps, qualitative research, telemedicine

## Abstract

**Background:**

There is a critical need to expand the pool of available kidneys for African Americans who are on the transplant wait-list due to the disproportionally lower availability of deceased donor kidneys compared with other races/ethnic groups. Encouraging living donation is one method to fill this need. Incorporating mHealth strategies may be a way to deliver educational and supportive services to African American transplant-eligible patients and improve reach to those living in remote areas or unable to attend traditional group-session-based programs. Before program development, it is essential to perform formative research with target populations to determine acceptability and cultivate a patient-centered and culturally relevant approach to be used for program development.

**Objective:**

The objectives of this study were to investigate African American kidney transplant recipients’ and kidney donors’/potential donors’ attitudes and perceptions toward mobile technology and its viability in an mHealth program aimed at educating patients about the process of living kidney donation.

**Methods:**

Using frameworks from the technology acceptance model and self-determination theory, 9 focus groups (n=57) were administered to African Americans at a southeastern medical center, which included deceased/living donor kidney recipients and living donors/potential donors. After a demonstration of a tablet-based video education session and explanation of a group-based videoconferencing session, focus groups examined members’ perceptions about how educational messages should be presented on topics pertaining to the process of living kidney donation and the transplantation. Questionnaires were administered on technology use and perceptions of the potential program communication platform. Transcripts were coded and themes were examined using NVivo 10 software.

**Results:**

Qualitative findings found 5 major themes common among all participants. These included the following: (1) strong support for mobile technology use; (2) different media formats were preferred; (3) willingness to engage in video chats, but face-to-face interaction sometimes preferred; (4) media needs to be user friendly; (5) high prevalence of technology access. Our results show that recipients were willing to spend more time on education than the donors group, they wanted to build conversation skills to approach others, and preferred getting information from many sources, whereas the donor group wanted to hear from other living donors. The questionnaires revealed 85% or more of the sample scored 4+ on a 5-point Likert scale, which indicates high degree of interest to use the proposed program, belief that other mHealth technologies would help with adherence to medical regimens, and doctors would make regimen adjustments quicker. In addition, high utilization of mobile technology was reported; 71.9% of the participants had a mobile phone and 43.9% had a tablet.

**Conclusions:**

Our study supports the use of an mHealth education platform for African Americans to learn about living donation. However, potential recipients and potential donors have differing needs, and therefore, programs should be tailored to each target audience.

## Introduction

### Overview

A barrier in the delivery of health education programs is the availability and proximity of the expert or professional to the intended audience, or patient. Technological advances provide opportunities to deliver health-related programs to disparate populations who lack transportation or time to attend sessions at a hospital, clinic, or other traditional health care settings. Educational programs traditionally consist of one-on-one conversations and group interactions where educators and learners can interact directly. Although telephone-based education can be used to reduce geographical barriers, it lacks the personal cues and nuances of in-person contact. Prerecorded video or audio clips can increase a learners’ knowledge about a subject, but they usually lack personalization or tailoring to individual needs [1]. Each medium presents a conundrum between costs and the intrinsic reach of the communication method [2].

Health education can now be disseminated worldwide through mobile health (mHealth) platforms, patient-provider telehealth (ie, videoconferencing), and video education using a multitude of Web services that have been increasing worldwide over the past decade [3]. With 92% of US adults owning a cellular phone irrespective of age, sex, or race, and 50-65% with access to a smartphone and/or a tablet, mHealth is a utilitarian opportunistic method for interventions [4,5]. In 2013, 21% of Americans had conducted video calls on cell phones or tablets, a proportion that is projected to grow substantially as smartphone adoption and access to such apps increase [6]. Many individuals use mHealth technologies to assist in making behavioral changes, but inconsistent results in clinical research signal the need for more formative research [4]. Failure to influence behavior change has in part been attributed to the lack of using behavioral theory and technology application models as a foundation for change and not appropriately contextualizing the designs of program apps, user interfaces, or educational content [7].

End-stage renal disease (ESRD) affects the lives of nearly 500,000 Americans [8] and is optimally treated with kidney transplantation. Kidney transplantation has become the gold-standard treatment, with multiple studies establishing its association with superior quality of life, improved life expectancy, and better psychosocial functioning, all at substantially lower cost than dialysis [9-12]. Unfortunately, the number of patients who need kidney transplants at a given point in time far exceeds the availability of deceased donor kidneys matching patients’ tissue type, especially among African American (AA) ESRD patients [13]. The number of living donors is not sufficient to close this gap in need. Increasing living donations is therefore an important health priority in this population. Furthermore, transplants from living donors result in longer and higher quality of life than deceased donor transplants [12,14]. AA ESRD patients experience a lower rate of living donor kidney transplantation compared with whites [15], which is associated with a lower level of engagement in the living kidney donation process [16]. Research suggests that AA ESRD patients are in need of greater support and education to heighten their opportunities to obtain a living donor kidney transplant [17].

### Living Kidney Donation Process

The typical living kidney donation process involves a brief group-educational class at a hospital setting in which ESRD patients and potential donors are informed about the medical screening the potential donor engages in to become eligible to donate a kidney, as well as the actual transplant procedures. However, states that include a large rural proportion that have few or only one transplant center, such as South Carolina, require other solutions to educate the populace. Lower national rates of living donor kidney transplantation in AAs compared with other ethnic groups [15] have been associated with lower knowledge levels about the process, as well as lower self-efficacy to discuss with others about considering to become a donor [18,19]. Several programs have successfully increased efforts to engage in the living donor kidney transplantation process and a few have increased rates of living donor assessments. These programs used transplant centers, home visits, and community meetings as the locations to build rapport and educate others about the living donor kidney transplantation process [20,21]. These types of personal contact methods may be replicated and made more accessible through technology when center or home visits are not feasible.

### Study Objective

The purpose of this study was to evaluate the attitudes, acceptance, and preferences of AA kidney transplant recipients, kidney transplant donors, and potential donors who learn about living kidney donation through an mHealth program. This study was designed to create a framework that will aid in the decision making and content delivery in support of such an intervention. We used a formative analytic approach to ensure that the program is culturally sensitive, patient centered, and conducive to different learning styles.

## Methods

### Research Design Overview

This study used a mixed-method design incorporating both qualitative and quantitative methods to assess the attitudes, perceptions, and user characteristics for a future mHealth living donor kidney transplantation program tailored for AA dialysis patients eligible for transplantation. This included use of focus groups with open-ended questions to enable targeted discussions and conversations about mHealth delivery preferences, as well as questionnaire data for quantitative analyses [22,23]. We assessed how technology might be used to educate individuals about living donor kidney transplantation, how such a future program might be designed, and how the contact and communication with the users should be organized. Items from the questionnaire were used to quantify use characteristics of mobile technology and attitudes toward a proposed tablet-enabled video module-based educational program in conjunction with group videoconferencing. Focus groups were used to qualitatively explore the context, perceptions, preferences, and scenarios for using mHealth-delivered education. The results of the study will inform the development of program materials for an mHealth educational delivery and group video chat program about living donor kidney transplantation education among AAs eligible for transplant.

### Development of Focus Group Questions

The technology acceptance model [24] and self-determination theory (SDT) [25] guided the development of the focus group interview questions. According to the technology acceptance model, two primary factors influence users’ decision as to how and when they will likely use a technology: perceived usefulness and perceived ease of use. These models were chosen to assess which mHealth technologies would be useful in an mHealth program (eg, group videoconferencing, video educational modules, text messaging, and email exchanges), as well as what features of the program may be needed to help motivate those targeted to initiate and continue using the program. In addition, multimedia learning theory [26] was used to frame and expand on the resultant themes to form recommendations for this sample and guide program development and communication for a living donor kidney transplantation program with AAs [24].

We selected SDT as the theoretical underpinning for the development of the program, as it is framed upon the process of fostering participation and sustaining involvement in behavioral change programs through development of competence (akin to self-efficacy in social cognitive theory [27]) and autonomous regulation. Consistent strong effects of these SDT mediators have been observed on sustained adherence to various health behavior programs (eg, smoking cessation, diet, physical activity) [28-31]. SDT conceptualizes a continuum of human motivational regulation, ranging from fully external to fully internal [25,28,30]. *External* regulation, a form of controlled motivation, includes extrinsic rewards and punishments administered by others. This would include, in addition to financial incentives/constraints, pressure from others to change (eg, family members, friends, health care providers). While external or controlled regulation may motivate change in the short term, such change is less enduring and less stable. The most autonomous form of motivation is internal or *autonomous regulation*. Here the person not only sees the importance of the behavior, but also links the change(s) with their other core values, beliefs, and life goals. Change arising from autonomous regulation is seen as the most stable and persistent [25,28,30]. Autonomy in SDT relates to our need to feel independent in our actions rather than feeling controlled or coerced.

The focus groups scripts were developed incorporating probe questions that identified how living donor transplant recipients developed competence and what factors helped them sustain their efforts to identify their donor. Transplant recipients who received a cadaver kidney provided input on how they may have experienced difficulties establishing competence in approaching others about living donation. Similarly, such information was obtained from the kidney donors and caregivers who were potential donors with regard to their involvement in going through the screening process for donation. In addition, probe questions were gathered from literature reviews and information gathered from prior AA kidney transplant study populations [13,16,18]. A team consisting of transplant surgeons, transplant coordinators, clinical psychologists, and experts in qualitative and quantitative methodology converged to develop the focus group questions.

### Questionnaire Selection

A 20-item questionnaire was selected to assess use of cellular technology and attitudes toward use of mHealth technology. The questionnaire was previously used in several studies that evaluated a prototype mHealth system for enhancing adherence to medical regimens with several different ethnic minorities (ie, hypertensive Hispanics, AA, and white kidney transplant recipients) [32,33]. A total of 11 questions used a yes/no response format and assessed patients’ access and utilization of mobile phones and mobile technologies, and awareness of telehealth programs (item content presented in Table 2). The remaining 9 items assessed respondents’ attitudes toward mHealth- and telehealth-based remote monitoring and used the following 5-point Likert item response format: 1=strongly disagree, 2=disagree, 3=neutral, 4=agree, and 5=strongly agree. Previous studies reported Cronbach alpha internal consistency coefficient of .92 for the 9 items [32,33]. As in previous studies [32,34], the questions were administered following a brief demonstration of an example of the mHealth video module prototype program. A brief video module (approximately 1.5 minutes) was presented on a tablet and addressed the topic of whether a donor had to be a blood relative. It utilized an approach of having a narrator introduce the particular topic domain and then led into a brief interview of a living donor and recipient. The video culminates in the narrator providing a brief summary (along with main points summarized and presented in a bullet framework while the narrator summarized each point). The transplant recipients, caregivers, and potential donors then completed the 9 items, which assessed their level of interest to use such a system if it was available when they were involved in the living donation process or if such a need ever arises again.

### Recruitment

Before recruitment, all research activities were reviewed and approved by the Medical University of South Carolina’s (MUSC) Institutional Review Board. Recruitment was performed by an AA transplant coordinator from MUSC. A list of living kidney transplant donors, potential donors (who were caretakers), living donor transplant recipients, and deceased donor transplant recipients were invited to participate in the focus groups by phone contact. Comparable numbers of participants were recruited with high-school education or less compared with those with advanced technical trade skills or college education level to ensure diverse education backgrounds in the sample.

### Study Implementation

A total of 9 focus groups were conducted and recorded in private conference rooms at MUSC. The focus groups were led by an AA nurse scientist who was a former kidney transplant coordinator and experienced in conducting focus groups. She had no prior contact with the patients and conducted the focus group protocol after obtaining verbal informed consent. Each focus group session started with introducing our concept and rationale for a future mHealth living kidney donor educational program. This was followed by a brief description of a program that would include weekly homework assignments of viewing brief educational video clips developed by AA living donor transplant recipients, AA living donors, transplant health care experts, etc, followed by weekly group videoconference sessions led by an AA living kidney donor transplant recipient. Participants were informed that the proposed group videoconferencing sessions would use a smartphone or tablet with the purpose of reviewing and expanding upon educational video clip assignments. Then, a demonstration of a prototype educational video clip about myths on living donor matching, including testimonials of an AA living donor transplant recipient and his donor was viewed by each focus group using a 10-in (25.4-cm) tablet. Afterward, group discussion commenced with the focus group questions (see Multimedia Appendix 1). At the end of the focus group questions, a questionnaire was given that took approximately 10 minutes to self-administer. A few participants preferred the questionnaire to be read to them. The focus group and completion of the subsequent questionnaire lasted approximately 60-90 minutes. Participants received a US $50 gift card at the end of the meeting to compensate them for their time and travel.

### Qualitative Analysis

The focus group audio recordings were transcribed verbatim by a professional transcription company, and the transcripts were uploaded to NVivo 10.0 (QSR International Pty, Doncaster, Victoria, Australia) for qualitative analysis. A directed content analysis was applied to the transcripts, applicable when existing theory guides the investigation [35]. Because the technology acceptance model [24] and SDT [25] guided the development of interview questions, our emphasis was to assess the roles of the underlying tenets of the technology acceptability model (eg, perceived value, easy access and utilization, easy means of rectifying technical problems) and theory (eg, competence in using system, motivation to sustain engagement*)*. This was framed in the context of the proposed mHealth-technology-enabled combination video module and group videoconferencing educational and motivational enhancement program. In this regard, 2 raters individually searched the transcripts for participants’ views on the use of the technology being proposed, its perceived usefulness, ease of use, and intention to use. These were coded to develop the final themes. Coding was conducted on 9 transcripts representing a total of 57 AA focus group participants who were either living kidney transplant donors or potential donors (ie, caregivers of ESRD patients, n*=*30) or were kidney transplant recipients (n*=*27). Two of the authors (JS and LN) independently read all transcripts; one of the authors (LN) coded these transcripts using NVivo. Immersion and crystallization [36] were used to validate the analysis. Two of the authors (JS and LN) examined patterns and themes in the data, and integrated them with the primary tenets of the technology acceptance model and SDT [37]. After the initial coding process [38], the 2 qualitative analysts (JS and LN) reviewed the coding results to crystallize the findings through an intensive review of the common themes. *Immersion* entailed examination of the data in detail, involving careful reading to absorb and inductively derive what was important in the transcribed and coded texts, and *crystallization* involved using these coded data to reflect on the analysis, query specific constructs, and identify the key themes noted in the immersion phase [36]. This produced another focused set of iterative codes in which both qualitative researchers reconciled the final themes. The use of NVivo software in the analysis produced an audit trail of the coding decisions, which ensures credibility of findings. The internal validity of findings was ensured using well-known methods for content analysis and the triangulation of the interview findings with the survey results. Iterative questions within the focus group guide were used to probe the participants, which provided the opportunity to corroborate and verify understanding by the interviewers [39].

### Quantitative Analyses

The 20-item questionnaire used Yes/No and Likert-scale responses and SPSS v20 (SPSS IBM, New York, NY, USA) was used for the analysis. Item responses were categorized into dichotomous groupings. The 11 cellular technology utilization and prior awareness of telehealth/mHealth questions were readily categorized as yes versus no. The 9 Likert-scale questions had 5 response options to understand the acceptability of the mHealth-enabled group-delivered educational/motivational program. The responses were placed into one of two categories: agree (from strongly agree to agree) versus neutral/disagree (ranging from neutral, disagree, to strongly disagree). Overall group percentages, as well as stratified percentages, were tabulated between transplant recipients and the living donor/potential donor groups. In addition, for the 9 items assessing attitudes toward the mHealth prototype program, stratified comparisons of responses were made to evaluate the potential influence of age (<50 versus ≥50 years) and education (high-school diploma or less versus more than high-school diploma, including technical/trade school*,* partial college, college graduate).

Independent *t* tests were used to compare differences between groups on continuous item responses (eg, age). Chi-square tests were used to examine group differences in responses to categorical data (eg, marital status, ethnicity; Table 1) and the 20 items assessing cellular utilization and attitudes toward mHealth conceptual program (Tables 2 and 3). Test statistics were reported with *P* values, with *P*<.05 considered significant.

**Table 1:** Demographics of the participants.

		All N=57	Transplant recipients N=27	Donors/potential donors N=30	*P* value
**Sex, n (%)**					.26
	Male	23/57 (40.4)	13/27 (48.1)	10/30 (33.3)	
	Female	34/57 (59.6)	14/27 (51.9)	20/30 (66.7)	
Age, mean (SD) years		46.7 (13.3)	48.6 (14.6)	45.0 (11.9)	.37
Years on dialysis before transplant, mean (SD)			2.8 (2.4)		
**Ethnicity, n (%)**					.34
	African American	56/57 (98.2)	27/27 (100.0)	29/30 (96.7)	
	Hispanic	1/57 (1.8)	0/27 (0.0)	1/30 (3.3)	
**Marital status, n (%)**					.49
	Married	31/57 (54.4)	12/27 (44.4)	17/30 (56.7)	
	Divorced/separated	10/57 (17.5)	6/27 (22.2)	6/30 (20.0)	
	Never married	14/57 (24.6)	8/27 (29.6)	6/30 (20.0)	
	Widowed	2/57 (3.5)	1/27 (3.7)	1/30 (3.3)	
**Education, n (%)**					.64
	College graduate	26/57 (45.6)	10/27 (37.0)	16/30 (53.3)	
	Trade/technical school; partial college	9/57 (15.8)	4/27 (14.8)	5/30 (16.7)	
	High-school diploma	20/57 (35.1)	12/27 (44.4)	8/30 (26.7)	
	Less than high-school diploma	2/57 (3.5)	1/27 (3.7)	4/30 (13.3%)	
**Annual income**					.18
	<US $15,000	10/57 (17.5)	8/27 (29.6)	2/30 (6.7)	
	US $15,001-29,999	10/57 (17.5)	5/27 (18.5)	5/30 (16.7)	
	US $30,000-49,999	13/57 (22.8)	4/27 (14.8)	9/30 (30.0)	
	US $50,000-75,000	8/57 (14.0)	3/27 (11.1)	5/30 (16.7)	
	>US $75,000	2/57 (3.5)	0/27 (0.0)	2/30 (6.7)	
	Prefer not to answer	13/57 (22.8)	7/27 (25.9)	7/30 (23.3)	
**Employment status, n (%)**					<.001
	On disability	17/57 (29.8)	14/27 (51.9)	3/30 (10.0)	
	Full time	25/57 (43.9)	5/27 (18.5)	20/30 (66.7)	
	Part-time	2/57 (3.5)	1/27 (3.7)	1/30 (3.3)	
	Retired	9/57 (15.8)	6/27 (22.2)	3/30 (10.0)	
	Unemployed	4/57 (7.0)	1/27 (3.7)	3/30 (10.0)	

## Results

### Demographics of Study Participants

A total of 57 individuals participated in 9 focus groups between January and March 2013. The demographics of the participants are reported in Table 1. Two groups each of living and deceased donor transplant recipients (n*=*27) and 2 groups each of living donors and potential donors (caretakers) of prior recipients (n*=*30) made up the sample. All were AA except 1 female living donor, who was Hispanic and married to an AA recipient. The overall age was 46.7 (SD 13.3) years with a range of 23-72 years. Most of the participants were married (54%, 31/57), with approximately equal numbers of those with a college education (46%, 26/57) versus those who had a trade-school education or less. A third of the sample (30%, 17/57) was on disability with 47% (27/57) holding full- or part-time employment. There was a wide range of reported personal income between US $15,000 and US $75,000, with a median income between US $30,000 and US $50,000—23% (13/57) of questionnaires were missing income data. With regard to employment status of the sample, 22% (6/27) of transplant recipients were employed at least part-time, compared with 70% (21/30) of the living donors/caregivers (*P*<.001).

### Questionnaire Findings

Several sets of comparisons were made on the questions assessing utilization of cellular technology and attitudes toward the tablet-delivered prototype demonstration of the conceptual educational/motivational enhancement program. The Cronbach alpha internal consistency coefficient was .95 for the items assessing attitudes toward the tablet-delivered prototype program. These results are comparable to previously reported Cronbach alpha coefficient of .92 [32,33]. No statistically significant differences were found between the transplant recipient and kidney donor/caregiver groups on the 11 questions that assessed cellular technology utilization and awareness of mHealth/telehealth (all chi-square *P*>.09; Table 2). Across the entire sample, most owned a cell phone (91%, 52/57) and 72% (41/57) regularly used a smartphone; 86% (49/57) used text messages, 70% (40/57) used email, and 79% (45/57) routinely accessed the Internet via their phones. After being given a definition of mHealth and telehealth, 74% (42/57) reported they had never heard of mHealth or telehealth concepts before the focus group. Tablets were prevalent in 44% (25/57) of households, and 95% (54/57) reported someone at home could offer assistance with using a smartphone or tablet if needed.

**Table 2:** Cellular technology-use questions delivered by questionnaire or read-out loud.

% of participants marked “Yes”	All	Transplant recipients	Donors/potential donors	*P* value (chi-square test)
1. Do you already have a working cellular phone?	52/57 (91)	24/27 (89)	28/30 (93)	.554
2. Do you already have a working “smartphone”-capable cellular device (Internet capable)?	41/57 (72)	17/27 (63)	24/30 (80)	.153
3. Does anyone in your household already have a working cellular phone?	52/57 (91)	23/27 (85)	29/30 (97)	.126
4. Does anyone in your household already have a working “smartphone”-capable cellular phone?	45/57 (79)	19/27 (70)	26/30 (87)	.131
5. Do you already have a working tablet computer like an iPad?	25/57 (44)	11/27 (41)	14/30 (47)	.653
6. If you need help with using your cellular phone or tablet, is there someone in your household who can help you?	54/57 (95)	26/27 (96)	28/30 (93)	.617
7. Send or receive text messages?	49/57 (86)	21/27 (78)	28/30 (93)	.091
8. Send or receive email?	40/57 (70)	19/27 (70)	20/30 (67)	.764
9. Use the Internet?	45/57 (79)	20/27 (74)	25/30 (83)	.392
10. Download ringtones or apps?	42/57 (74)	18/27 (67)	24/30 (80)	.253
11. Have you heard of telehealth or mobile health before today?	15/57 (26)	5/27 (19)	10/30 (33)	.205

Following the focus group discussion and demonstration of the prototype video module, participants rated their attitudes toward the conceptual program discussed using the Likert-scale response format from 1 (strongly disagree) to 5 (strongly agree). Table 3 presents the percentage rates of the 5-item format responses placed within 2 categories, namely, agree (strongly agree or agree) or disagree (neutral/disagree/strongly disagree). Across the entire sample, all scores trended toward acceptability with an agree rating (≥80%). For example, there was a high level of acceptability in being educated remotely by health care providers via technology (93%, 53/57). The entire sample believed that mHealth programs would enable them to both receive information quickly from health care providers (89%, 51/57) and communicate with them when needed (91%, 52/57).

**Table 3:** Attitudes toward mHealth technology and proposed living donor transplant education program by categories of participants, age and education.

% that marked strongly agree or agree	All^a^	Recipients^a^	Donors and potential donors^a^	Type of participant *P* value (chi-square test)	Age<50 years^a^	Age≥50 years^a^	Age *P* value (chi-square test)	High-school diploma or less^a^	More than high-school diploma^a^	Education *P* value (chi-square test)
Would use mHealth devices if free	49/57 (86)	23/27 (85)	26/30 (87)	.872	31/34 (91)	20/23 (87)	.610	15/22 (68)	34/35 (97)	.002
If someone available to answer questions likely to use devices as directed	50/57 (88)	24/27 (89)	26/30 (87)	.800	32/34 (94)	18/23 (78)	.074	17/22 (77)	33/35 (94)	.057
Comfortable having health monitored remotely by doctor/nurses using mHealth technologies	53/57 (93)	26/27 (96)	27/30 (90)	.352	33/34 (97)	21/23 (91)	.340	20/22 (91)	34/35 (97)	.305
Comfortable using cell phone	51/57 (90)	25/27 (93)	26/30 (87)	.466	32/34 (94)	21/23 (91)	.683	18/22 (82)	34/35 (97)	.046
mHealth technology will help remind me to follow doctor’s directions	50/57 (88)	24/27 (89)	26/30 (87)	.800	32/34 (94)	21/23 (91)	.683	19/22 (86)	34/35 (97)	.121
mHealth technology could allow my doctor to make medication changes quicker	51/57 (90)	24/27 (89)	27/30 (90)	.891	31/34 (91)	21/23 (91)	.986	19/22 (86)	33/35 (94)	.303
Confident my privacy is protected when using mHealth devices	47/57 (83)	24/27 (89)	23/30 (77)	.226	33/34 (97)	20/23 (87)	.143	18/22 (82)	33/35 (94)	.135
Important to follow doctor’s directions	54/57 (95)	26/27 (96)	28/30 (93)	.617	33/34 (97)	21/23 (91)	.340	20/22 (91)	34/35 (97)	.305
Confident mHealth technology can effectively communicate my medical condition to my doctor	52/57 (92)	26/27 (96)	26/30 (87)	.199	33/34 (97)	21/23 (91)	.340	20/22 (91)	34/35 (97)	.305

^a^All values are presented as n/N, %.

Importantly, there were high levels of comfort in using phone- or tablet-delivered video educational modules and participating in group videoconference sessions with others in the same circumstances (89%, 51/57). The lowest level of acceptability (82%, 47/57) involved their level of confidence that their use of video modules and statements made during videoconference chat sessions would remain secure over the Internet.

We also evaluated the potential modulating influence of age and education on the participants’ attitudes. As shown in Table 3, although there were no statistically significant differences between younger and older participants, there was a trend for those aged under 50 to be more likely to engage in mHealth device utilization if technical assistance was available. Level of education was associated with attitudes toward mHealth technology. Participants having high-school diploma or less felt less facile in using a cell phone (*P*=.046), were less likely to use mHealth technology even if provided for free (*P*=.002), and if technical assistance was readily available (*P*=.057). Similar to the entire cohort, there were nonsignificant trends indicating that those aged over 50 years having high-school diploma or less were less confident that their comments over videoconference sessions, etc would remain secure over the Internet.

### Qualitative Findings

The following 5 major themes were found to be consistent among most of the focus group participants.

1. Few reservations to use mobile technology
2. Different media formats would be nice to have
3. Willingness to engage in video chats, but face-to-face meetings were sometimes preferred
4. Media needs to work quickly and be easy to use
5. Access to technology help within their immediate circles

**Figure 1:**
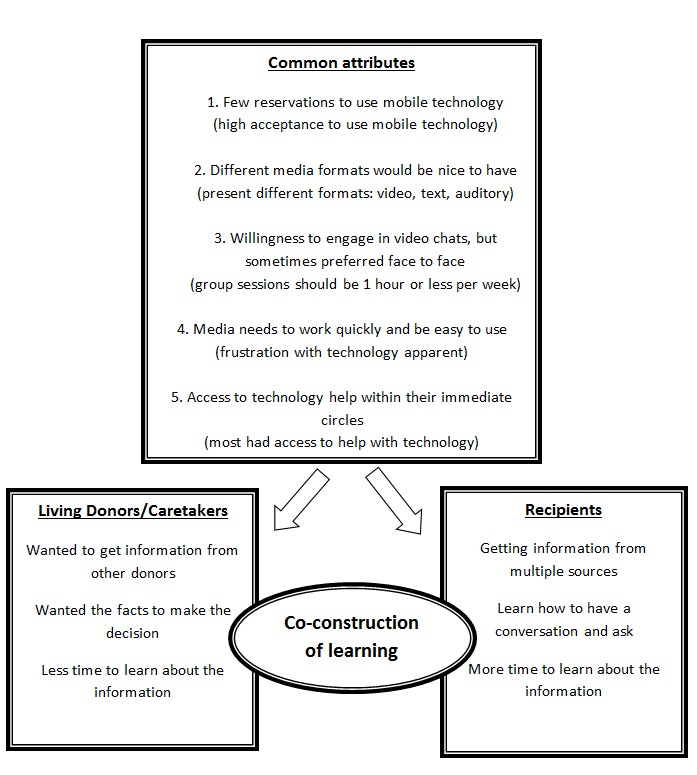
Similarities and differences between kidney transplant donors/potential donors and transplant recipients in mHealth-based preferences in learning about living kidney donor transplantation.

There were distinct information needs and preferences between the prior kidney donor/potential donor and the prior kidney transplant recipient groups but there were also many common attributes that were shared from the 5 major themes (Figure 1). It was clear that there should be a co-construction of learning together with others in the same situation to be able to overcome barriers regarding lack of information about the living donor kidney transplantation process. Co-construction of learning places emphasis on learning together, fostering increased clarity, and understanding of others’ experiences, perspectives, and viewpoints on the issues under consideration [40]. Potential donors who were typically family members who serve as caretakers and former transplant donors retrospectively voiced preferring to have received information from other donors. They preferred to learn this information in a concise format so that they would be better equipped to make their decision regarding kidney donation. By contrast, prior transplant recipients (both living and deceased donors) preferred getting information from multiple sources including others like them in addition to medical professionals. They expressed potential benefits from exposure to learning how to have a conversation with potential donors, address questions they may have, and make the request for a kidney donation.

### Few Reservations in Use of Mobile Technology

Overall, the participants were familiar with mobile technology such as smartphones and tablets. Participants discussed enjoying video clips as a way to learn quickly from others. Several stated video clips were preferred. Some commented,

I mean the video clip would be good and just put a bit of, not much they have to read, but just enough to know what is going on. A little, just a little video clip and a little note of what’s going on.

If I had a choice, then a video is what I would like.

For some people it would be easier to watch a video; it is easier to sit there with some headphones on and just listen to what is being read on the screen.

### Different Media Formats Would Be Nice to Have

Others pointed out that text messaging provided helpful reminders and motivators in other health-related programs they experienced. One participant commented,

I would kind of mix it up a little bit, because, like I just registered myself with this Mommy and Me text messages that I get to my phone daily about my pregnancy, and you know they’ll be sending me text messages every day about ultrasounds, and to make sure you are eating the right foods, so the iPads plus the text messages to me would, they would both help.

### Willingness to Engage in Video Chats, but Face-to-Face Meeting Is Sometimes Preferred

It was noted that there was enthusiasm for having experience in using a tablet (eg, iPad), and with that the willingness to try video chats as it at least provided a way to see the person that you are talking with. Face-to-face meeting was sometimes preferred, but the willingness to try new media was clear from the majority.

With the iPad you said you can do video chats, email, you can set it up where you can call and talk to the person on the phone. I figured, okay, if you can setting up reading and talk to them on the phone, you can always ask, hey can we go meet at Starbucks (coffee shop) or something and have a one-on-one conversation with each other if you're like in the same area or you can, to me you can always, like we have this focus group now, see if you can set up a group like that where everybody can meet together and also share their stories or their concerns about the process.

And as far as like time, you know, if you're super-super busy and your schedules will never link up for you to talk to this person face-to-face, if you have the iPad or something, whenever you do get a moment, you just get the information you need and probably get some of your questions answered that way.

Like if you say you are watching it in another hospital or whatever, just having that direct contact with that person that I can say, I can touch you and see that you are right here and I can ask you questions or whatever. I think that's important.

You're talking about an application like Skype or some other interactive. That would help.

I think it would depend on the person because some people they may have questions that they would be uncomfortable asking someone, you know, face-to-face that they don't really know, unless it's somebody you are comfortable with.

The opportunity to talk with a person who has had the experience was very appealing:

Anything else, it helps if you talked to somebody that's already been there and done that, you're better prepared. Nothing will shock you or, you know, you already know what you're getting into, the beginning, the middle, and end.

Yeah, to talk to somebody that's been there, been through it. That-that would help.

### Media Needs to Work Quickly, Be Short, and Easy to Use

It was clear that to some of the participants the bandwidth of Internet connections might be an issue that limits the effectiveness of using video.

I don't think video clips are for me, I don't really click on the video unless it's seriously something like I know for a fact I wanna sit down and watch. I have like slow Internet...a video I'm not as apt to click on it or wait for it to load or do whatever. Just a video clip, like yesterday I was like, okay, it was almost like, it was only two minutes long, but still.

### Access to Technology Help Within Their Immediate Circles

Many of the participants had resources that could help with technology barriers through family members, and local stores that offered technical support.

Sometimes there is, especially if they have like a young niece, nephew, grandchild around. If it's an older person like that, or you can always, I've noticed like Best Buy and Office Max and some of those places, they have it where you can come into their establishment with a iPad or a cell phone and the Geek Squad or somebody will help you get the hang of how to use it.

My grandbaby knows how to do all of that stuff and she ain't but nine and I don't know how.

## Discussion

### Principal Findings

In this study, we assessed rates of AA mobile phone ownership including smartphones and tablets, utilization of the devices’ features, awareness of mHealth technology, and attitudes toward use of video modules and group videoconferencing for living kidney donation education. This study identified several themes relevant to designing mHealth programs for potential AA kidney transplant recipients and donors. The focus group members were very familiar with smartphone and tablet technology even if they did not own one. From our findings, there were few reservations in using technology to disseminate education materials. This was especially evident in using brief video education clips to bring up topics and then using video chat sessions to discuss these points more fully. Although the lack of face-to-face content may be a factor in someone choosing to enroll in a program, there was an overall attitude of “Let’s try it.” Therefore, any program that integrates discussion using such means needs to be clearly defined and tested for the intelligibility of the conversation.

Our qualitative analyses-derived themes support using mobile communication technology in a living donor transplant education program. With increased access to smartphone technologies [5,41], there are fewer concerns with adopting mobile devices for use in programs. Participants who did not own a mobile device were still familiar with them and if supplied, endorsed that they would use them in a program. In addition, if help was needed, there was confidence in the groups that they knew someone who had a similar device to aid them if needed.

The concept of using different media formats is not new [42]. However, combining different methods to communicate messages concurrently may lead to less understanding and confusion [7,26]. For example, cognitive overload, described in learning theory, states that an excessive increase in cognitive load may occur if one has too many teaching methods concurrently presented such as an animation, voice delivery, and text on a video education clip. This is thought to be distracting and may mask the main learning point for those learning new concepts [24]. Limiting the number of communication styles may help in directing focus on the education topic of importance and is helped by cleaning up excessive information [7]. Different education delivery methods may cater to different learning styles, and therefore, reiterating the main concepts using a different delivery tactic, such as when summarizing information, may enhance learning.

There have been several successful programs that instituted face-to-face learning sessions for living donor education, as well as other approaches [2,43]. Rodrigue et al [43] showed an increase in living donor knowledge, patient identification of potential donors, and potential donor engagement in medical evaluations using a home-based visit engaging a transplant-eligible patient and his/her family members and friends in learning about living donation [43]. Although such face-to-face educational approaches were preferred by some in our sample, sending medical staff for home visits across the state or having patients travel to the single transplant medical center in South Carolina or other states with a large service area is not always convenient, easy, or cost effective to arrange. This would be especially noted for those on dialysis with financial constraints, and/or reliance on others for transportation. Many telehealth programs have been used in the past to assess patients’ medical status and provide education and medical regimen management with various chronic conditions [44]. The majority of these systems have used traditional telephone communication or desktop computer videoconferencing but with increased access of mobile communication technology in the consumer space, the interface can now be performed using consumer electronic devices such as smartphones and tablets.

Another important theme noted was the usability of the technology. It was important to our sample (participants) that the individual modules and overall program be created in such a way that it is easy to navigate to increase their perceived competence in using the program*,* as well as help motivate them to sustain using it [24,28,29]. Although smartphone and tablet use was commensurate with the national prevalence rates at the time, not all participants had a data plan through their cellular provider with allowable bandwidth or capacity to accommodate use of video streaming or group videoconferencing. There was concern on how such a program using a large amount of bandwidth or video would affect monthly service fees. A primary solution is to have the participants access Wi-Fi connectivity; therefore, a data plan is not required and assuring good streaming rate quality. Other solutions include use of lower bit-rate streaming videos optimized for mobile devices using online media distributors or embedding the videos into the app itself if broadband speeds or cellular data networks are too slow.

Although there were many common attributes between the groups, there were noted differences between kidney transplant recipients and living donors/potential donors with regard to preferences for who is going to deliver the video module educational messages. The kidney transplant donors/potential donors mostly wished to receive information from those who had been through the process, whereas the transplant recipients suggested receiving information from multiple sources. The kidney donors/potential donors were also interested in receiving a program’s educational modules as quickly as possible and required just the facts to make their decision as quickly as possible. The transplant recipient group differed, preferring to have more time to learn about the living donor transplantation process. In addition, the transplant recipients wanted skill-building learning modules that included passive and active modeling sessions with the transplant recipient navigator and other participants in the program before attempting such activities with family members and/or friends. Other studies have found that transplant-eligible patients benefit from such types of behavioral skill-building sessions [18]. It was also noted that the groups wanted to learn together with those in the same situation and walk through the process and support one another showing they were interested in colearning. This provides an interesting perspective for future studies to provide a social learning setting and may motivate sustained adherence to the programs.

### Limitations

Several limitations should be mentioned when interpreting the findings. The focus groups were made up of AA kidney transplant recipients, donors, and potential donors. These findings are context specific and may not be generalized to other ethnic groups or age groups. The sample was also taken from the coastal area of South Carolina and may not be representative of the immediate southeastern states or the rest of the country. Participants may have reacted to the focus group questions with an expectancy bias and given more positive responses due to the study placement conditions. Participants also may have self-selected to be part of the study and may have had a more positive attitude toward living donation when compared with others who may have not had transportation or time to meet for the focus group.

It should be noted that nonsignificant trends were observed, which indicated that the kidney transplant recipients were less likely than the kidney donors/potential donors to own a smartphone, use text messaging, and to have someone in the home with a smartphone. They were also less likely to be employed and as a result tended to earn less than the donors/potential donors. In addition, we did not formally evaluate the impact of limited cellular data plans (or lack of) with regard to acceptability of the proposed tablet-enabled program. However, as noted earlier, our likely approach to address such issues will be to use Wi-Fi connectivity, which eliminates the need for a data plan.

Although there were high levels of acceptability across participants toward the use of mHealth-technology-enabled programs, level of education and age showed modulating influence on some issues. Participants with high-school diploma or less were less comfortable using a cell phone and reported being less likely to use mHealth technology even if it was free or technical assistance was readily accessible. Similar to individuals aged over 50, those with high-school diploma or less were less confident that their comments during videoconference sessions, personal health data, etc would be kept secure across the Internet. Given the limited sample sizes of these subgroups of AA kidney transplant recipients, transplant donors, and potential donors, further research is warranted with these subgroups*,* as well as transplant-eligible patients to better understand potential barriers and challenges in the application of mHealth-enabled technologies in future transplant education programs.

Our study’s findings support use of tablet-based video educational modules and remote group discussions with an AA living kidney organ recipient as a navigator among AAs eligible for kidney transplantation. This study demonstrates the importance of how patients with chronic diseases are willing to spend more time learning about solutions to their disease compared with those who do not have the disease and could possibly be a potential donor preferring a quicker educational process. Although there was some apprehension in using distance-based group video chat sessions, the sample was optimistic about trying it out. This said, educational message delivery must be succinct and to the point for potential donors, but should be elaborated on for those who need a transplant. This formative research provides insight to be used when tailoring materials for AA populations on issues of living donation. Creative context-specific, mostly brief, segments of video-based education and communication should be developed to include similar people to the patient group who share their life struggles to address educational needs in potential AA kidney donors and recipients. The preliminary findings may spur development of innovative and cost-efficient mobile communication strategies, enhance willingness to adopt these strategies in educating target populations, aid in understanding how these platforms can be used, and determine the preferences of AA ESRD and potential donor populations in receiving these educational materials [13].

## Appendix

Multimedia Appendix 1 Focus group questions.

## Abbreviations

AA: African American
ESRD: end-stage renal disease
MUSC: Medical University of South Carolina
SDT: self-determination theory
