# Supplementary material for: Patient-Centered mHealth Living Donor Transplant Education Program for African Americans: Development and Analysis
Source: JMIR Res Protoc. 2015 Aug 10;4(3):e84. doi: 10.2196/resprot.3715 (PMC4705021; doi:10.2196/resprot.3715)
Supplement: Multimedia Appendix 1 [file resprot_v4i3e84_app1.pdf]

Appendix 1 Focus group questions

(donors/caregivers)

a. So In general, what do you think of the overall idea of a telemedicine system using the iPad to provide education and encouragement for potential kidney donors living throughout South Carolina?

b. If you were asked to consider being a donor, would it help you in making a decision of whether or not to become a donor? If not, why not?

c. Are you concerned about the lack of in-person contact involved with this system?

d. If not able to manage it yourself, do you have someone who could help assist you with the technology? *[Do you think most people have someone available? If not, any suggestions]*

e. Given all that we've discussed this evening what issues have we forgotten to include which will be important for patients to feel comfortable to approach others about considering being a donor?

f. Tell me what you think about having someone who has successfully donated a kidney help navigate (guide) other potential donors through the process…like a buddy system?

g. What about using the iPad?

h. Would it be best to use just brief video clips, or combinations of video clips, just auditory messages, options to read text, see charts, graphs. We will train patients how to use them.

i. How much time do you think you could spend looking at video clips, educational messages, etc. per week on the free iPad?

j. How long should the weekly session with the navigator and other patients last? We plan to have weekly sessions the first month and then a session twice a month for 2 months.

k. Finally, what suggestions do you have for new components or improvements? What kinds of things would be useful in providing education?

l. Finally, what suggestions do you have for new components or improvements? Probe: What have we missed?

(recipients)

a. So In general, what do you think of the overall idea of a telemedicine system using the iPad to provide education and encouragement for living donor transplant for patients across South Carolina?

b. Would you see yourself as a patient needing a kidney using this system if it is available to you? If not, why not?

c. Do you think this system would have helped you learn more about living donor kidney transplant?

d. Are you concerned about the lack of in person contact involved with this system?

e. How confidential do you think your information will remain with this system?

f. If not able to manage it yourself, do you have someone who could help assist you with the technology?

g. Given all that we've discussed this evening what issues have we forgotten to include which will be important for patients to feel comfortable to approach others about considering being a donor?

h. Tell me what you think about having someone who has successfully received a living donor kidney help navigate (guide) patients through the process…like a buddy system?

i. What about using the iPad?

j. Would it be best to use just brief video clips, or combinations of video clips , just auditory messages , options to read text , see charts, graphs, We will train patients how to use them .

k. How much time do you think you could spend looking at video clips, educational messages, etc. per week on the free iPad?

l. We plan to have weekly sessions the first month and then a session twice a month for 2 months. How long should the weekly session with the navigator and other patients last?

m. Finally, what suggestions do you have for new components or improvements? Probe: What have we missed?
